# Functional Characterization of the Transcription Factor Gene *CgHox7* in *Colletotrichum gloeosporioides*, Which Is Responsible for Poplar Anthracnose

**DOI:** 10.3390/jof10070505

**Published:** 2024-07-21

**Authors:** Qiuyi Huang, Fuhan Li, Fanli Meng

**Affiliations:** 1 Beijing Key Laboratory for Forest Pest Control, College of Forestry, Beijing Forestry University, Beijing 100083, China; hqy7313920632021@163.com (Q.H.); lifuhan@bjfu.edu.cn (F.L.); 2 The Key Laboratory for Silviculture and Conservation of Ministry of Education, College of Forestry, Beijing Forestry University, Beijing 100083, China

**Keywords:** *Colletotrichum gloeosporioides*, *CgHox7*, appressorium formation, pathogenicity

## Abstract

*Colletotrichum gloeosporioides* is the main pathogen that causes poplar anthracnose. This hemibiotrophic fungus, which can severely decrease the economic benefits and ecological functions of poplar trees, infects the host by forming an appressorium. Hox7 is an important regulatory factor that functions downstream of the Pmk1 MAPK signaling pathway. In this study, we investigated the effect of deleting *CgHox7* on *C. gloeosporioides*. The conidia of the Δ*CgHox7* deletion mutant germinated on a GelBond membrane to form non-melanized hyphal structures, but were unable to form appressoria. The deletion of *CgHox7* weakened the ability of hyphae to penetrate a cellophane membrane and resulted in decreased virulence on poplar leaves. Furthermore, deleting *CgHox7* affected the oxidative stress response. In the initial stage of appressorium formation, the accumulation of reactive oxygen species differed between the Δ*CgHox7* deletion mutant and the wild-type control. Moreover, *CgHox7* expression was necessary for maintaining cell wall integrity. Considered together, these results indicate that CgHox7 is a transcription factor with crucial regulatory effects on appressorium formation and the pathogenicity of *C. gloeosporioides*.

## 1. Introduction

Poplar anthracnose is an important tree disease that is widely distributed across China. With the extensive cultivation of poplar trees in recent years, the associated increase in poplar anthracnose caused by *Colletotrichum gloeosporioides* has resulted in widespread leaf withering and significant economic losses, while also decreasing the beneficial effects of poplar trees on ecosystems [1,2,3]. *C. gloeosporioides* is a typical hemibiotrophic fungus. After adhering to the host leaf surface, one side of the conidium germinates, forming a germ tube that develops into a mature appressorium. Subsequently, the appressorium generates a strong turgor pressure and forms a penetration peg that ruptures the host cuticle [4]. The penetration peg differentiates into primary and secondary hyphae that kill mesophyll cells, leading to the development of necrotic lesions, which are a hallmark symptom of anthracnose [5].

Various pathways regulating the pathogenicity-related processes of *C. gloeosporioides* (e.g., conidial germination, appressorium formation, and penetration) have been identified, including the mitogen-activated protein kinase (Pmk1 MAPK), cyclic adenosine monophosphate (cAMP), and target of rapamycin (TOR) pathways [6,7,8]. These pathways form a complex regulatory network responsive to fungal growth and infection-related processes in diverse environments [9]. This network primarily involves the regulation of gene expression and cellular differentiation by transcription factors. For example, in *C. gloeosporioides*, the Pmk1 MAPK pathway genes *CgSte50*, *CgSte12*, *CgSte11*, *CgSte7*, and *CgPmk1* have been functionally characterized. Deleting *CgPmk1* leads to a complete lack of appressorium formation and a loss of pathogenicity [10], whereas deleting *CgSte50*, *CgSte11*, and *CgSte7*, which function upstream of the Pmk1 MAPK pathway, results in the formation of defective appressoria and a loss of pathogenicity [11]. The deletion of *CgSte12*, which functions downstream of the Pmk1 MAPK pathway, does not substantially alter vegetative growth and appressorium formation, but it affects pathogenicity [12].

Homeobox-domain-containing proteins, which are ubiquitous among animals, plants, and fungi, are characterized by their homeobox-DNA-binding motifs that enable them to function as transcription factors controlling the expression of multiple genes. Hox7, which is encoded by a homeobox gene family member, is conserved in animals, plants, and fungi, wherein it plays a crucial role in morphogenesis [13]. In *Magnaporthe oryzae*, MoHox7 is a critical regulatory factor that functions downstream of the Pmk1 MAPK signaling pathway and must be phosphorylated by Pmk1 to coordinate the expression of genes related to appressorium formation and development [14]. Deleting *MoHox7* abolishes appressorium formation, with germ tubes elongating abnormally (several rounds of swelling and hooking), leading to decreased pathogenicity. MoHox7 regulates cell cycle progression and autophagy during appressorium development [2], effectively suppressing hyphal growth and appressorium differentiation [14]. Similarly, in *Colletotrichum orbiculare*, the homolog of *Hox7* (i.e., *Hox3*) influences appressorium formation and affects pathogenicity on host leaves [13]. However, transcription factors in diverse pathogenic fungi regulate the metabolism of cellular substances, leading to the formation of various infection structures (e.g., attachment cells), resulting in differences in the development time and the appearance of symptoms on the infected host (e.g., lesions), making them species-specific and environment-specific factors influencing infections.

In this study, we identified and functionally characterized *CgHox7* in *C. gloeosporioides* on the basis of analyses of phenotypes and gene expression. Our findings indicate that deleting *CgHox7* results in decreased appressorium formation and virulence because of the associated changes to cell wall integrity, oxidative stress responses, and the accumulation of reactive oxygen species (ROS) during conidial germination. The study results provide a theoretical basis for developing strategies to control poplar anthracnose pathogens and for identifying potential fungicide targets.

## 2. Materials and Methods

### 2.1. Fungal Strains and Culture Conditions

A wild-type (WT) *C. gloeosporioides* strain CFCC80308 was isolated from *Populus × beijingensis* in Beijing, China [15] and used to generate the Δ*CgHox7* and Δ*CgHox7/HOX7* mutants. The WT and mutant strains were maintained on solid potato dextrose agar (PDA) medium at 25 °C to induce sporulation. Liquid complete medium (CM) was used for culturing mycelia, which were collected to extract DNA, whereas TB3 medium (3 g yeast extract, 3 g casamino acids, 20% sucrose, and 0.7% agar) was used to screen for deletion mutants.

### 2.2. Bioinformatics Analysis of CgHox7

The CgHox7 sequence (Protein ID: EVM0009219.1) of CFCC80308 strain (genome sequence data have been deposited in GenBank, accession numbers: PRJNA961031) was obtained by screening the *C. gloeosporioides* genome database (http://genome.jgi.doe.gov/Gloci1/Gloci1.home.html, accessed on 3 June 2023) using BLASTP, with the *C. gloeosporioides Cg-14* Hox7 sequence (EQB44263-13294) serving as a query. The CgHox7 amino acid sequence was determined on the basis of the homology to amino acid sequences in other fungi, which was revealed by BLASTP. In addition, a phylogenetic tree was constructed using MEGA 6.0 along with full-length protein sequences and the neighbor-joining method (1000 bootstrap replicates). CgHox7 domains were predicted using the InterProScan tool (https://www.ebi.ac.uk/interpro, accessed on 3 June 2023).

### 2.3. Targeted Gene Knockout and Complementation

A published split-marker method [16] was used to construct a deletion cassette containing the hygromycin B resistance gene (*Hph*) to replace the native *CgHox7* gene in WT *C. gloeosporioides*. To generate the *CgHox7* deletion mutant, approximately 1.6 kb upstream and downstream flanking sequences were amplified by PCR using the primer pairs CgHox7-5Ffor/CgHox7-5Frev and CgHox7-3Ffor/CgHox7-3Frev. The *Hph* cassette was amplified by PCR using the primer pair hygromycinfor and hygromycinrev, which include an approximately 20 bp sequence that overlaps (M13F or M13R) the two flanking sequences amplified by PCR. After a fusion PCR, the upstream and downstream fragments were fused with two-thirds of the *Hph* cassette. The Δ*CgHox7* mutant was produced via a PEG-mediated transformation as described previously [11]. The fusion fragments were mixed with the protoplasts of WT *C.gloeosporioides* in PEG buffer solution for 12–14 h to obtain the transformants. The resulting transformants were initially selected using TB3 medium supplemented with 300 μg/mL hygromycin B. Individual transformants successfully grown were recovered from the selective medium and cultured on the PDA medium for DNA extraction. The mutations were confirmed by performing a PCR analysis using the primers External-CgHox7for/External-CgHox7rev and Internal-CgHox7for/Internal-CgHox7rev.

The complementation mutant was generated by co-transforming Δ*CgHox7* mutant protoplasts with the full-length *CgHox7* coding sequence with the 1.6 kb upstream region and a phleomycin resistance cassette (amplified from the pBC-phleo vector provided by FGSC) according to a PEG-mediated transformation method, as described in Wang et al. [11]. Transformants were first selected using TB3 medium containing 200 μg/mL phleomycin, with mutations confirmed by PCR using the primers Internal-CgHox7for/Internal-CgHox7rev. All primers used in this study are listed in Table S1.

### 2.4. Conidial Germination and Appressorium Formation

Selected strains were cultured on solid PDA medium in plates for 5 days, after which the surface of the medium in each plate was washed repeatedly with sterile water to obtain a conidial suspension. A blood cell counting plate was used to adjust the conidial suspension concentration to 2 × 10^4^ conidia/mL. Equal volumes (30 μL) of conidial suspensions from each strain were added to the hydrophobic surface of a GelBond membrane for a 12-h culture at 25 °C in a constant temperature incubator (12.5 cm filter paper soaked in sterile water to maintain humidity). Samples were examined using an optical microscope and photographed after 12 h. All experiments were repeated three times.

### 2.5. Cellophane Membrane Penetration Assay

To conduct penetration assays, cellophane membranes were cut into 3 × 3 cm squares and autoclaved at 120 °C for 20 min. Sterile cellophane membranes were placed in the center of solid PDA medium in plates, after which hyphal blocks of each strain were placed on top of the cellophane membrane for 3 days. The cellophane membrane was removed and then the new colonies that grew on the PDA medium were examined and photographed 2 days later. The penetration assay was repeated three times.

### 2.6. Pathogenicity Assay

Leaves of the susceptible species *Populus* × *beijingensis* were used to evaluate the pathogenicity of the WT and mutant *C. gloeosporioides* strains. Current-year poplar branches were maintained in water for 2 weeks before leaves were collected. The surface of intact leaves was inoculated with equal volumes (30 μL) of conidial suspensions (2 × 10^5^ conidia/mL) or 5 mm hyphal blocks. The inoculated leaves were placed in Petri dishes containing moistened filter paper and sterile cotton wool. The Petri dishes were incubated at 25 °C in a constant temperature incubator. All leaves were examined for disease symptoms and photographed at approximately 8 days post-inoculation. Necrotic lesions were measured using quadrille paper. The pathogenicity assay was repeated three times.

### 2.7. Stress Sensitivity Assay

The WT, Δ*CgHox7*, and Δ*CgHox7/HOX7* strains were grown on solid PDA medium for 4 days and then 5 mm hyphal blocks (collected at the colony edges) were used to inoculate fresh solid PDA medium containing 100 μg/mL Congo red (CR), 1.2 M NaCl, 1 M sorbitol, or 5 or 10 mM H_2_O_2_ (final concentrations) in plates. After a 4-day incubation in a constant temperature incubator set at 25 °C, each colony was photographed. The stress sensitivity assay was repeated three times.

### 2.8. ROS Staining Assay

The WT, Δ*CgHox7*, and Δ*CgHox7/HOX7* strains were cultured on solid PDA medium in plates for 5 days, after which the medium surface was washed repeatedly with sterile water to obtain a spore suspension (2 × 10^4^ conidia/mL). The conidial suspensions (30 μL) were added to the hydrophobic surface of a GelBond membrane moistened with 12.5 cm filter paper soaked in sterile water for a 4- or 8-h culture at 25 °C. Next, 50 μL nitroblue tetrazolium (NBT) solution (0.05%) was added dropwise to the GelBond membrane for a 2-h staining at 25 °C in a constant temperature incubator. Samples were examined using an optical microscope and photographed. The ROS staining assay was repeated three times.

## 3. Results

### 3.1. Characterization and Deletion of CgHox7 in C. gloeosporioides

**Figure 1:**
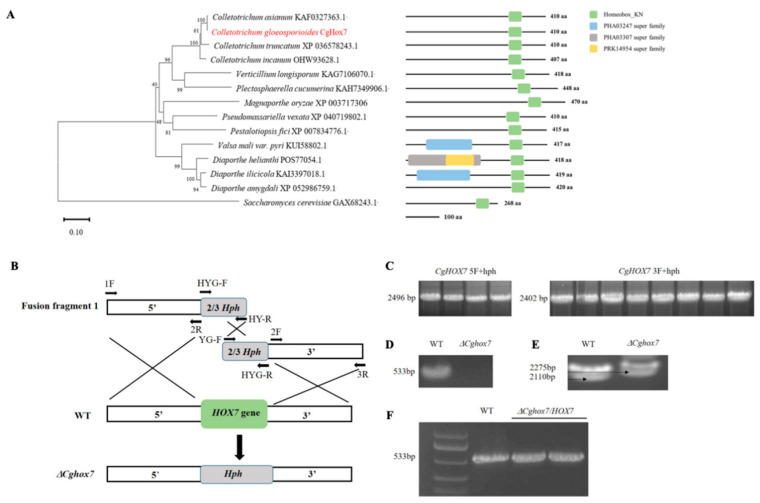
Phylogenetic analysis of CgHox7 and orthologs and deletion of *CgHox7*. (**A**) A neighbor-joining phylogenetic tree was constructed according to the amino acid sequences of representative fungal homeodomain transcription factors and CgHox7; domains were predicted using the InterProScan 5.0 tool. The numbers at nodes represent the percentage of their occurrence in 10,000 bootstrap replicates. (**B**) Schematic diagram of the *CgHox7* knockout vector constructed using the split-marker method. (**C**) Agarose gel electrophoresis analysis of upstream and downstream flanking sequences fused with two-thirds of the *Hph* cassette. (**D**) *CgHox7* deletion mutant verified by a PCR amplification using internal primers. (**E**) *CgHox7* deletion mutant verified by a PCR amplification using external primers. (**F**) *CgHox7* complementary strain verified by a PCR amplification using internal primers.

CgHox7 (Protein ID: EVM0009219.1) was identified by a BLASTP search of the *C. gloeosporioides* genome database (http://genome.jgi.doe.gov/Gloci1/Gloci1.home.html, accessed on 3 June 2023) using the *C. gloeosporioides Cg-14* Hox7 sequence (EQB44263-13294) as a query. A phylogenetic analysis of CgHox7 and orthologs in other fungi revealed the close relationship between *C. gloeosporioides* and *Colletotrichum asianum*. A homeobox domain was detected in CgHox7 (Figure 1A). To functionally characterize *CgHox7*, it was replaced with *Hph* via PEG-mediated transformation (Figure 1B–E). The full-length *CgHox7* sequence was reintroduced into the Δ*CgHox7* mutant to produce the complementation mutant (Figure 1F).

### 3.2. CgHox7 Does Not Regulate Hyphal Vegetative Growth

**Figure 2:**
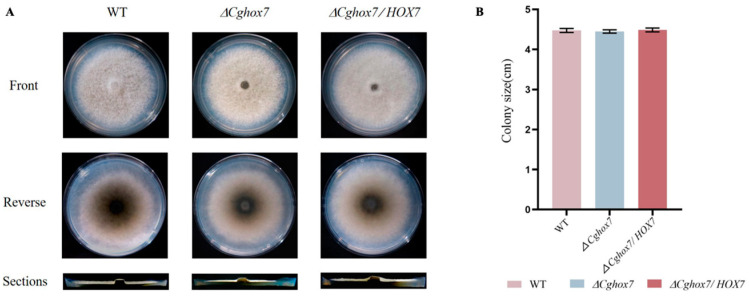
Effect of deleting *CgHox7* on *C. gloeosporioides* vegetative growth. (**A**) Colonies of WT, Δ*CgHox7*, and Δ*CgHox7/HOX7* strains grown on PDA medium in plates at 25 °C for 4 days. The lower panel presents the front, back, and side of the colonies of the indicated strains. (**B**) Bar chart of the colony sizes of the strains presented in (**A**). Error bars represent the standard deviation.

To investigate the effect of *CgHox7* on *C. gloeosporioides* vegetative growth, WT, Δ*CgHox7*, and Δ*CgHox7/HOX7* strains were grown on solid PDA medium for 4 days. In terms of their colony diameters, there were no major differences in the vegetative growth of the three strains (Figure 2A). The PDA medium in plates was cut longitudinally to examine the growth status and aerial hyphae thickness of each strain (Figure 2A), which revealed a lack of significant differences among the three strains (Figure 2B). These findings suggest that *CgHox7* does not affect *C. gloeosporioides* hyphal growth.

### 3.3. CgHox7 Is Essential for Appressorium Formation and Maturation

**Figure 3:**
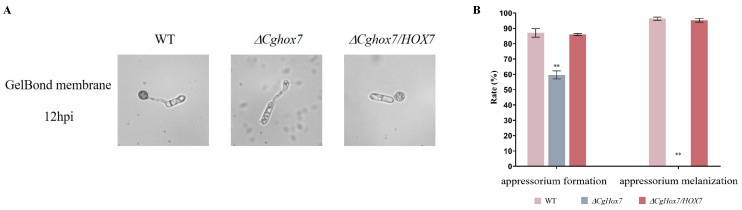
Regulatory effects of *CgHox7* on appressorium formation and maturation. (**A**) Conidia of the WT, Δ*CgHox7*, and Δ*CgHox7/HOX7* strains germinated on the hydrophobic surface of a GelBond membrane. (**B**) Bar chart of the appressorium formation rate and appressorium melanization rate for the strains presented in (**A**). Data were analyzed using Duncan’s multiple range test. ** indicate statistically significant differences (*p* < 0.05).

During an infection of susceptible plants, *C. gloeosporioides* forms an appressorium, which is a specialized infection structure. In this study, the WT strain formed numerous appressoria on the hydrophobic surface of a GelBond membrane (appressorium formation rate of 87%) at 12 h post-inoculation (hpi), with a melanization rate of 96.3%. The appressorium formation rate (86%) and melanization rate (95.2%) of the Δ*CgHox7/HOX7* complementation strain did not differ significantly from the corresponding WT rates. In contrast, at 12 hpi, only some of the Δ*CgHox7* mutant conidia germinated to form an appressorium (59.7%), while the rest generated slender hyphal structures (Figure 3A,B). The appressoria of the Δ*CgHox7* mutant were significantly smaller than those of the WT strain and were not melanized, indicating that they were in the early appressorium formation stage (Figure 3B). These results imply that *CgHox7* plays a crucial role in regulating appressorium formation and maturation.

### 3.4. CgHox7 Regulates the Penetration Ability of Hyphae

**Figure 4:**
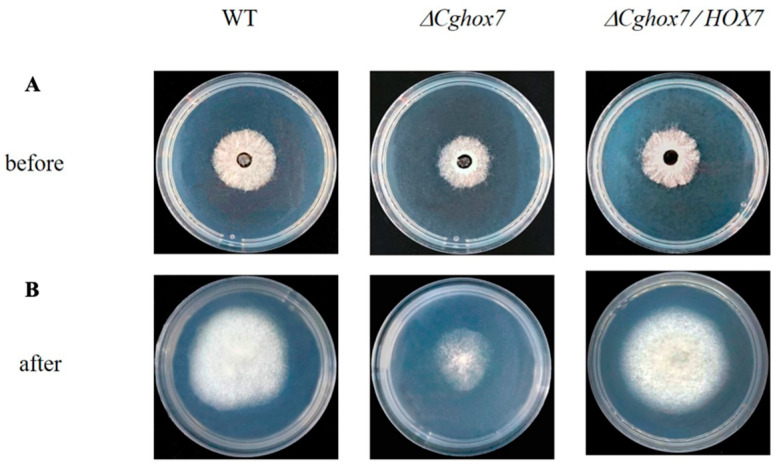
Cellophane membrane penetration assay. Three strains were grown on cellophane membranes overlaid on solid PDA medium in plates for 3 days at 25 °C ((**A**) before). After the cellophane membrane was removed, the plates were incubated at 25 °C for 2 days ((**B**) after).

To determine whether *CgHox7* influences the penetration ability of hyphae, the surface of a cellophane membrane placed on top of solid PDA medium in plates was inoculated with the WT, Δ*CgHox7*, and Δ*CgHox7/HOX7* strains and then incubated at 25 °C for 3 days (Figure 4A). The cellophane membrane was removed and the plates were maintained at 25 °C for an additional 2 days (Figure 4B). Although all three strains were able to penetrate the cellophane membrane, the WT control and the Δ*CgHox7/HOX7* complementation strain grew significantly more than the Δ*CgHox7* mutant on the PDA medium (Figure 4B). Therefore, *CgHox7* is likely involved in regulating the ability of *C. gloeosporioides* mycelia to penetrate certain materials (e.g., plant host tissue).

### 3.5. Disruption of CgHox7 Results in a Loss of Virulence

**Figure 5:**
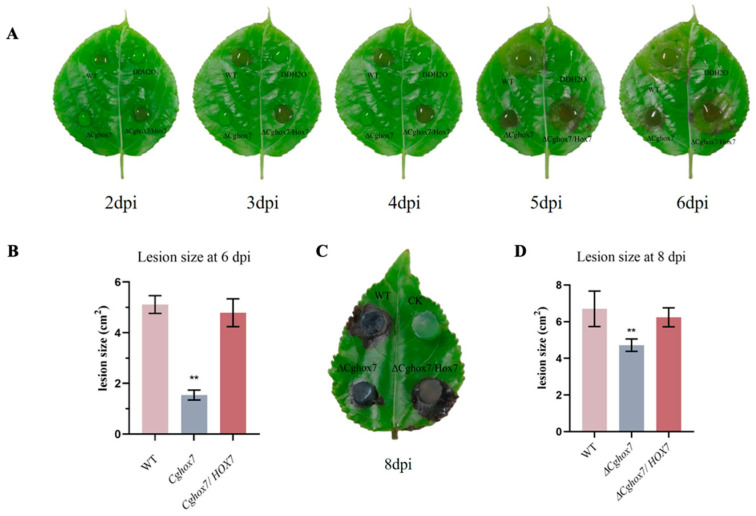
Pathogenicity assay on poplar leaves. (**A**) Intact leaves were inoculated with equal volumes (30 µL) of conidial suspensions (2 × 10^5^ conidia/mL) from WT, Δ*CgHox7*, and Δ*CgHox7/HOX7* strains and then incubated at 25 °C under humid conditions. The inoculated leaves were photographed at 2–6 dpi. (**B**) Bar chart of the lesion sizes of leaves 6 days after being inoculated with WT, Δ*CgHox7*, and Δ*CgHox7/HOX7* conidial suspensions. Error bars represent the standard deviation. Data were analyzed using Duncan’s multiple range test. ** indicate statistically significant differences (*p* < 0.05). (**C**) Intact leaves were inoculated with 5 mm hyphal blocks of WT, Δ*CgHox7*, and Δ*CgHox7*/*HOX7* strains and then incubated at 25 °C under humid conditions. The inoculated leaves were photographed at 8 dpi. (**D**) Bar chart of the lesion sizes of leaves 8 days after being inoculated with WT, Δ*CgHox7*, and Δ*CgHox7/HOX7* hyphal blocks. Error bars represent the standard deviation. Data were analyzed using Duncan’s multiple range test. ** indicate statistically significant differences (*p* < 0.05).

To further investigate the potential effects of *CgHox7* on pathogenicity, intact poplar leaves were inoculated with WT, Δ*CgHox7*, and Δ*CgHox7/HOX7* conidial suspensions (2 × 10^5^/mL). At 2–4 days post-inoculation (dpi), necrotic lesions formed and spread at a steady rate on the leaves inoculated with the WT or Δ*CgHox7/HOX7* conidial suspension, whereas lesions were undetectable on the leaves inoculated with the Δ*CgHox7* conidial suspension (Figure 5A). The decreased virulence of the Δ*CgHox7* mutant may be related to its weakened ability to penetrate the leaf surface. However, at 5 dpi, necrotic lesions were detected on the leaves inoculated with the Δ*CgHox7* conidial suspension (Figure 5A,B). Intact leaves were also inoculated with WT, Δ*CgHox7*, and Δ*CgHox7/HOX7* hyphal blocks. At 1–8 dpi, the lesions on the leaves inoculated with the Δ*CgHox7* hyphal block expanded more slowly than the lesions on the leaves inoculated with the WT or Δ*CgHox7/HOX7* hyphal block (Figure 5C,D), providing additional evidence of the decreased virulence of the Δ*CgHox7* mutant. These results indicate that *CgHox7* is required for the full virulence of *C. gloeosporioides*.

### 3.6. CgHox7 Is Required for Cell Wall Integrity

**Figure 6:**
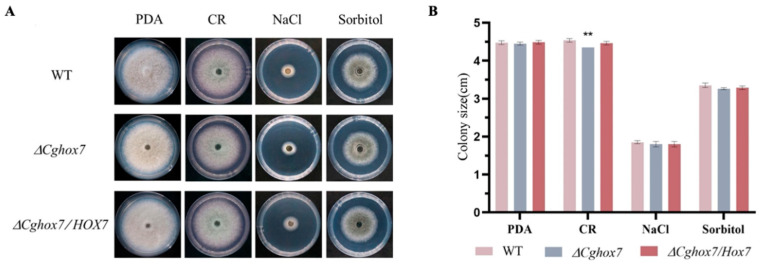
Responses to abiotic stresses. (**A**) Hyphal blocks of the WT, Δ*CgHox7*, and Δ*CgHox7/HOX7* strains were used for the inoculation of solid PDA medium containing 100 μg/mL CR, 1.2 M NaCl, or 1 M sorbitol (final concentrations) in plates, which were photographed at 4 dpi. (**B**) Bar chart of the colony sizes of the strains on solid PDA medium and solid PDA medium containing 100 μg/mL CR, 1.2 M NaCl, or 1 M sorbitol. Error bars represent the standard deviation. Data were analyzed using Duncan’s multiple range test. ** indicate statistically significant differences (*p* < 0.05).

A cell wall inhibitor and osmotic stressors were used to determine whether *CgHox7* contributes to abiotic stress responses. More specifically, PDA medium containing 100 μg/mL CR, 1.2 M NaCl, or 1 M sorbitol was inoculated with the WT, Δ*CgHox7*, or Δ*CgHox7/HOX7* strain (Figure 6A). The growth rate of these three strains on medium containing 1.2 M NaCl or 1 M sorbitol was determined. There were no major differences in the inhibitory effects of 1.2 M NaCl or 1 M sorbitol on growth among the Δ*CgHox7*, WT, and Δ*CgHox7/HOX7* strains (Figure 6B). Thus, *CgHox7* may not be critical for the osmotic stress response of *C. gloeosporioides*. However, compared with the WT and Δ*CgHox7/HOX7* strains, the Δ*CgHox7* strain differed significantly in terms of vegetative growth on PDA medium supplemented with 100 μg/mL CR (Figure 6A,B). This result indicates that *CgHox7* may be involved in maintaining cell wall integrity.

### 3.7. Deletion of CgHox7 Affects the Oxidative Stress Response

**Figure 7:**
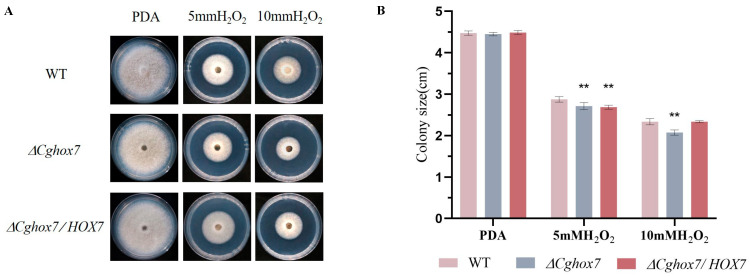
Response to oxidative stress induced by H_2_O_2_. (**A**) Hyphal blocks of the WT, Δ*CgHox7*, and Δ*CgHox7/HOX7* strains were used for the inoculation of solid PDA medium or solid PDA medium containing 5 or 10 mM H_2_O_2_ in plates, which were photographed at 4 dpi. (**B**) Bar chart of the colony sizes of the strains under H_2_O_2_-induced oxidative stress conditions. Error bars represent the standard deviation. Data were analyzed using Duncan’s multiple range test. ** indicate statistically significant differences (*p* < 0.05).

In plants, ROS can directly inhibit the growth of pathogens, thereby providing protection against infections [17]. The rapid accumulation of ROS at the infection site is one of the earliest responses to a pathogen attack. Therefore, a successful infection of a plant host by pathogenic fungi depends on an appropriate oxidative stress response. To determine whether *CgHox7* is involved in the response to oxidative stress, we inoculated solid PDA medium containing 5 or 10 mM H_2_O_2_ with hyphal blocks of the WT, Δ*CgHox7*, and Δ*CgHox7/HOX7* strains. At 4 dpi, the colony sizes of the three strains were recorded (Figure 7A). The 10 mM H_2_O_2_ treatments inhibited the growth of the Δ*CgHox7* strain significantly more than the growth of the WT and Δ*CgHox7/HOX7* strains (Figure 7B), possibly reflecting the importance of *CgHox7* for the oxidative stress response of *C. gloeosporioides*.

### 3.8. ROS Accumulation during Conidial Germination Is Affected by the Deletion of CgHox7

**Figure 8:**
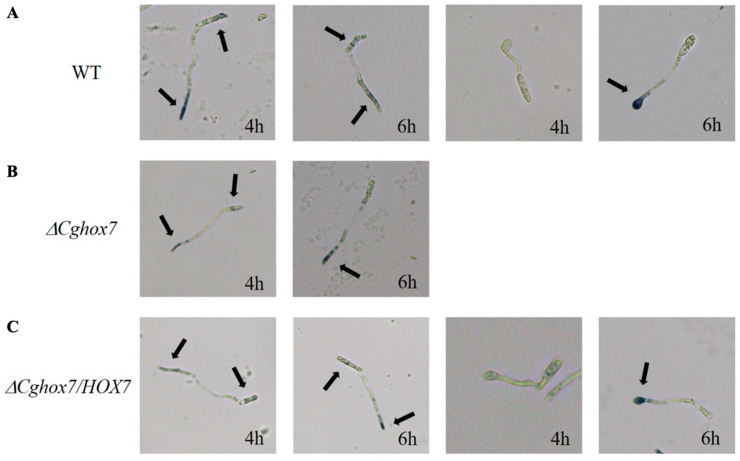
Distribution of intracellular ROS during conidial germination. The hydrophobic surface of a GelBond membrane was inoculated with equal volumes (30 μL) of conidial suspensions (2 × 10^4^ conidia/mL) from (**A**) WT, (**B**) Δ*CgHox7*, and (**C**) Δ*CgHox7/HOX7* strains. Water was replaced with 30 μL 0.05% (*w*/*v*) NBT dissolved in 0.05 M sodium phosphate, pH 7.5, at 4 and 6 hpi. After 2 h, the reaction was stopped by replacing the NBT solution. Black arrows indicate the accumulation of intracellular ROS (blue precipitate).

Plant pathogenic fungi produce ROS during conidial germination and appressorium formation [18,19]. To assess whether deleting *CgHox7* disrupts the accumulation of endogenous ROS during conidial germination, the initial stage of *C. gloeosporioides* appressorium formation was analyzed by NBT staining, which can reveal the presence of superoxide ions (detected as blue precipitates) [20]. At 4 hpi, endogenous ROS were mainly distributed at the anterior part of the conidia and germ tubes in the WT and Δ*CgHox7/HOX7* strains. The WT and Δ*CgHox7/HOX7* strains had partially formed appressoria at 4 hpi, but there was relatively little accumulation of ROS in the appressoria and conidia (Figure 8A,C). At the same time point, the Δ*CgHox7* strain lacked appressoria. Moreover, ROS accumulated mainly at the anterior part of germ tubes, with a smaller amount within the conidia (Figure 3B). At 6 hpi, ROS also accumulated at the anterior part of WT conidia and germ tubes that had yet to form an appressorium. Most of the WT germ tubes eventually formed appressoria, which accumulated relatively large amounts of ROS (Figure 8A). Similar results were obtained for the Δ*CgHox7/HOX7* complementation strain (Figure 8C). However, the Δ*CgHox7* strain still lacked appressoria, with ROS mainly concentrated at the anterior part of germ tubes (Figure 8B). These observations indicate that the distribution of endogenous ROS is disrupted during the germination of Δ*CgHox7* conidia.

## 4. Discussion

Homeobox transcription factors are highly conserved in eukaryotes. Notably, their functions have been systematically characterized in several fungi. For example, *Hox7*, which is a homeobox gene family member, encodes an important regulator of fungal morphology [21]. In the current study, we determined that CgHox7 in *C. gloeosporioides* is a crucial transcription factor for regulating appressorium formation, cellophane membrane penetration, pathogenicity, cell wall integrity, oxidative stress response, and ROS accumulation during conidial germination.

In *M. oryzae*, MoHox1–8 are transcription factors significantly affecting functional development and pathogenicity. Mutations to the genes encoding these transcription factors reportedly decrease vegetative growth (Δ*Mohox1* and Δ*Mohox6* mutants), disrupt conidial development (Δ*Mohox2* and Δ*Mohox4* mutants), or result in no observable phenotypic changes (Δ*Mohox3* and Δ*Mohox5* mutants); however, the Δ*Mohox7* mutant cannot produce appressoria and is non-virulent on intact leaves, but can still grow within plant cells, whereas the Δ*Mohox8* mutant can form appressoria, but is non-pathogenic because of a defective osmotic stress response [2]. In the present study, the Δ*CgHox7* mutant was normal in terms of vegetative growth of hyphae and conidial development (Figure 2 and Figure 3), but the inability of its appressorium to form normally ultimately resulted in decreased penetration and virulence (Figure 4 and Figure 5).

The melanization of germinated conidia expressing *HOX7* differs between *C. gloeosporioides* and *M. oryzae*. A previous study showed the *M. oryzae* Δ*Mohox7* mutant fails to develop appressoria, but forms germ tubes that elongate abnormally during several rounds of swelling and hooking [2]. The *C. orbiculare* Δ*Cohox3* mutant (deletion of a *CgHox7* homolog) also lacks appressoria, but its conidia germinate to produce lightly pigmented structures that are smaller than WT appressoria and have elongated hyphae [13]. The conidia of the Δ*CgHox7* mutant germinated on a GelBond membrane and host leaves, but only 59.7% formed an appressorium at 12 hpi; this appressorium formation rate was significantly lower than the corresponding rate for the WT strain. Moreover, the appressoria were smaller than the WT appressoria and were not melanized, which are characteristics associated with the early stage of appressorium formation (Figure 5). On the basis of this observation combined with the delayed pathogenicity and decreased virulence (i.e., relatively small lesions) of the Δ*CgHox7* mutant (Figure 5), we speculate that the deletion of *CgHox7* may have detrimental effects on appressorium formation and development.

Pmk1 MAPK, which is homologous to the yeast MAPKs Fus3/Kss1, is important for regulating the differentiation of the mature appressorium and invasive hyphae [22,23,24]. Successive phosphorylation of upstream MAPKKs (Ste11 and Ste7) mediates the activation of MAPK cascades in filamentous fungi, and subsequently activated Pmk1 MAPK can phosphorylate downstream substrates and regulate various functions [25,26]. Earlier research showed that the *M. oryzae* infection of plants mediated by appressoria is regulated by Pmk1 MAPK and hierarchical transcriptional networks dependent on the related transcription factors Hox7 and Mst12. MoHox7, which is directly targeted by Pmk1 MAPK, is the most critical transcription factor associated with *M. oryzae* appressorium morphogenesis. Its phosphorylation by Pmk1 MAPK regulates autophagy and cell cycle arrest to promote appressorium development [14]. Additionally, Mst12, which also functions downstream of Pmk1 MAPK, is responsible for regulating appressorium maturation and repolarization. The Δ*Momst12* mutant is apparently normal in terms of vegetative growth, conidial germination, appressorium formation, and melanization; however, it is non-pathogenic on rice leaves and exhibits defective growth [27]. The *C. gloeosporioides* Δ*Cgste12* (*MST12*) mutant is phenotypically similar to the Δ*Momst12* mutant [12]. Therefore, we speculate that there is also a hierarchical network of transcription factors (including Hox7 and Mst12) regulated by Pmk1 MAPK or additional transcription factors in *C. gloeosporioides*. Notably, the interaction between Hox7 and Mst12 remains unclear.

In this study, we also revealed the effect of *CgHox7* on abiotic stress responses and ROS distribution during appressorium development. Fungus-specific Zn_2_Cys_6_ transcription factor genes are involved in regulating growth, asexual development, conidial germination, appressorium formation, pathogenicity, and stress responses [28]. Among these genes, *FZC64* and *FZC52* are directly regulated by Hox7 [14]. In *M. oryzae*, both of these genes contribute to multiple stress responses [28]. Therefore, the deletion of *CgHox7* likely prevents the activation of Fzc64 and Fzc52. However, in our study, compared with the WT strain, the Δ*CgHox7* mutant differed significantly in terms of cell wall integrity and oxidative stress response; it was also unaffected by the 1.2 M NaCl and 1 M sorbitol treatments (Figure 6 and Figure 7). We speculate that there are other transcription factors that control *FZC64* and *FZC52* expression or other genes associated with osmotic stress responses. Following an infection by pathogenic fungi, plants rapidly release ROS as a defense mechanism [17]. In addition, an oxidative burst at the infection site is one of the earliest plant defense responses. Therefore, the adaptation of pathogenic fungi to oxidants is critical for their virulence. The Δ*CgHox7* mutant was adversely affected by oxidants, which may help to explain its decreased virulence (Figure 7). Interestingly, while infecting a plant host, pathogenic fungi also produce ROS derived from NADPH oxidase during conidial germination and appressorium formation. In eukaryotes, ROS are key signaling molecules that can modify signaling proteins to activate the MAPK pathway [29,30,31]. In our study, the deletion of *CgHox7* weakened the ability of the mutant to form appressoria, suggesting that the production of endogenous ROS during conidial germination may have been disrupted. Unsurprisingly, at 6 hpi, the Δ*CgHox7* mutant still lacked appressoria and ROS continued to accumulate in the anterior part of the germ tube; these characteristics differed significantly from those of the WT strain (Figure 8). Earlier research showed that the thioredoxin gene *TRX2* affects intracellular ROS signaling in *M. oryzae*. Specifically, Trx2 functions upstream of the Pmk1 MAPK pathway; the deletion of the corresponding gene leads to a decrease in the phosphorylation levels of Pmk1 [5]. Therefore, we propose that there is a certain regulatory relationship between *CgTRX2* or other unidentified redox-related genes and CgHox7, which may be associated with the observed changes in ROS accumulation in the Δ*CgHox7* mutant. Future studies will need to elucidate the relationships among intracellular ROS, Pmk1 MAPK, and CgHox7 as well as the underlying molecular mechanisms (Figure S1).

In conclusion, our study revealed the importance of the transcription factor CgHox7 for *C. gloeosporioides* appressorium formation, appressorium melanization, penetration, pathogenicity, cell wall integrity, oxidative stress response, and ROS accumulation. Our results clarified the pleiotropic effects of CgHox7 in *C. gloeosporioides* and suggest that CgHox7 may be a key regulatory factor affecting Pmk1 MAPK.

## Data Availability

Data are contained within the article or Supplementary Materials.

## Supplementary Materials

The following supporting information can be downloaded at: https://www.mdpi.com/article/10.3390/jof10070505/s1. Figure S1: The hypothetical model about Hox7 in Pmk1 MAPK of *C. gloeosporioides*; Table S1: Primers used in this study.
