# Supplementary material for: Functional Characterization of the Transcription Factor Gene CgHox7 in Colletotrichum gloeosporioides, Which Is Responsible for Poplar Anthracnose
Source: J Fungi (Basel). 2024 Jul 21;10(7):505. doi: 10.3390/jof10070505 (PMC11278219; doi:10.3390/jof10070505)
Supplement: Supplementary file 1 [file jof-10-00505-s001.zip › jof-3065347-supplementary.pdf]

Table S1 Primers used in this study.

Primer Name	Sequence
CgHOX7-5Ffor	TGGCACGTTGGATAGTCAGT
CgHOX7-5Frev	AATGGTGTGGGCTCGAAAAC
CgHOX7-3Ffor	AACTACGATCGGGACCTGAC
CgHOX7-3Frev	CCTCAGCCAAACAACAGTCC
External-CgHOX7for	ACCTCACCATCACATCAGCA
External- CgHOX7rev	CATTTGTCTGCCCACCATCC
Internal- CgHOX7for	AGAGTGAGAGAGTTAGCCGC
Internal- CgHOX7rev	CTTGCGTTGTTTGCTGTCAC
HPH-F	CGCCAGGGTTTTCCCAGTCACGAC
HPH-R	AGCGGATAACAATTTCACACAGGA
HY-R	GATGTAGGAGGGCGTGGATATGTCCT
YG-F	GATGTAGGAGGGCGTGGATATGTCCT

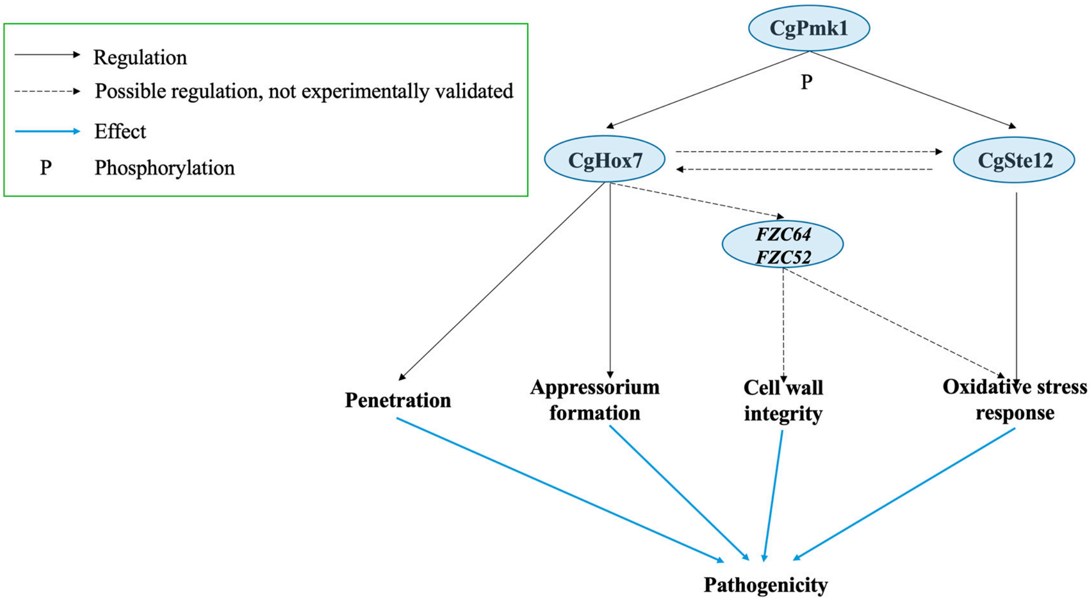

Figure S1 The hypothetical model about Hox7 in Pmk1 MAPK of *C. gloeosporioides*.
